# Performance Evaluation of Machine Learning Methods for Leaf Area Index Retrieval from Time-Series MODIS Reflectance Data

**DOI:** 10.3390/s17010081

**Published:** 2017-01-01

**Authors:** Tongtong Wang, Zhiqiang Xiao, Zhigang Liu

**Affiliations:** State Key Laboratory of Remote Sensing Science, Beijing Normal University, Beijing 100875, China; ttwang@mail.bnu.edu.cn (T.W.); zhigangliu@bnu.edu.cn (Z.L.)

**Keywords:** BPNN, GRNNs, leaf area index, RBFNs, MSVR, retrieval

## Abstract

Leaf area index (LAI) is an important biophysical parameter and the retrieval of LAI from remote sensing data is the only feasible method for generating LAI products at regional and global scales. However, most LAI retrieval methods use satellite observations at a specific time to retrieve LAI. Because of the impacts of clouds and aerosols, the LAI products generated by these methods are spatially incomplete and temporally discontinuous, and thus they cannot meet the needs of practical applications. To generate high-quality LAI products, four machine learning algorithms, including back-propagation neutral network (BPNN), radial basis function networks (RBFNs), general regression neutral networks (GRNNs), and multi-output support vector regression (MSVR) are proposed to retrieve LAI from time-series Moderate Resolution Imaging Spectroradiometer (MODIS) reflectance data in this study and performance of these machine learning algorithms is evaluated. The results demonstrated that GRNNs, RBFNs, and MSVR exhibited low sensitivity to training sample size, whereas BPNN had high sensitivity. The four algorithms performed slightly better with red, near infrared (NIR), and short wave infrared (SWIR) bands than red and NIR bands, and the results were significantly better than those obtained using single band reflectance data (red or NIR). Regardless of band composition, GRNNs performed better than the other three methods. Among the four algorithms, BPNN required the least training time, whereas MSVR needed the most for any sample size.

## 1. Introduction

Leaf area index (LAI) is defined as one half the total green leaf area per unit horizontal ground surface area [1]. It is an important biophysical parameter, and is widely used in crop growth monitoring, yield estimation, and global change studies. Currently, many methods have been developed to retrieve LAI from remote sensing data. These inversion methods can be generally divided into two categories: statistical methods and physical methods [2]. The statistical methods employ the statistical relationships between vegetation indices (VIs) and LAI to estimate LAI [3,4,5]. The physical methods are based on the inversion of radiative transfer models that clearly describe the connection between biophysical variables and canopy reflectance [6,7,8]. Because the physical methods can be adjusted for a wide range of situations [9], radiative transfer models are increasingly used in the inverse mode to estimate LAI from remotely sensed data. However, the commonly used inversion techniques based on iterative minimization of a cost function require excessively computationally demanding in order to achieve high retrieval accuracy [9]. For operational applications, machine learning methods based on precomputed radiative transfer simulations [10] or existing LAI products [11,12] are popular inversion techniques.

Among the machine learning methods, artificial neural networks (ANNs) are the most widely used techniques to retrieve LAI from remote sensing data [13,14]. Danson et al. [15] used a canopy radiative transfer model to simulate reflectance data for a wide range of biophysical conditions and these data were used to train back-propagation neural networks (BPNN) to retrieve LAI from measured reflectance data. The results showed that the estimation of sugar beet LAI using the trained neural network is more reliable than the LAI values retrieved using statistical relationships between VIs and LAI. Fang et al. [16] used ANN methods to retrieve LAI from the Landsat-7 Enhanced Thematic Mapper Plus surface reflectance and top of atmosphere radiance, respectively, and the results showed that the ANN methods can estimate LAI accurately. Bacour et al. [17] designed a multilayer perceptron to estimate LAI, the fraction of absorbed photosynthetically active radiation (FAPAR), the fraction of vegetation cover (FCover), and the canopy chlorophyll content (LAI × Cab) from Medium Resolution Imaging Spectrometer (MERIS) surface reflectance data. Baret et al. [10] developed an algorithm to generate global LAI, FAPAR, and FCover products from VEGETATION observations based on training BPNN with SAIL + PROSPECT radiative transfer model simulations for each biophysical variable. The GEOV1 LAI retrieval algorithm relies on BPNNs trained with the “best estimates” of LAI obtained by fusing and scaling the Moderate Resolution Imaging Spectroradiometer (MODIS) and CYCLOPES LAI products and the VEGETATION nadir surface reflectance values over the Benchmark Land Multisite Analysis and Intercomparison of Products (BELMANIP) sites [11]. Zhu et al. [18] used feedforward neural networks trained with the best-quality MODIS LAI data to generate LAI products from the third-generation Global Inventory Modeling and Mapping Studies (GIMMS3g) normalized difference vegetation index (NDVI) data.

Support vector machines (SVMs) are another type of popular machine learning method and are well known for their good performance in classification and function approximation. In recent years, SVMs are increasingly being used for the estimation of biophysical variables from satellite observations. Durbha et al. [19] proposed a method for LAI retrieval using support vector regression (SVR) from multiangle imaging spectroradiometer data. The method used a single-output SVR and could retrieve only one parameter at a time. Multi-output support vector regression (MSVR) [20,21] expanded single-output SVR into multiple-output and used ε-insensitive cost function to maintain simple and sparse solution. Tuia et al. [22] used MSVR for estimation of LAI × Cab, LAI and FCover from hyperspectral compact high-resolution imaging spectrometer images.

Indeed, many machine learning methods have been used to estimate LAI from remotely sensed data. On the one hand, most machine learning methods use satellite observations at the specific time to retrieve LAI, a result of which is that the LAI products generated by these methods are spatially incomplete and temporally discontinuous [23]. To generate a long-time series of high-quality global LAI product, Xiao et al. [12] developed an operational algorithm for estimating LAI from time-series MODIS surface reflectance data using general regression neural networks (GRNNs) with a multi-input-multi-output architecture. This method achieved excellent performance and was used to produce the Global Land Surface Satellite (GLASS) LAI product from time-series MODIS and AVHRR surface reflectance data [24]. On the other hand, each machine learning method has its own pros and cons, and there is no performance comparison between the machine learning methods for LAI retrieval from time-series remote sensing data.

In this study, machine learning algorithms were developed to retrieve LAI from time-series MODIS surface reflectance data. The reprocessed MODIS reflectance data for a one-year period were entered into the trained machine learning algorithms to estimate LAI profiles for the one-year period. The performance of the machine learning algorithms was evaluated by retrieving LAI from time-series MODIS reflectance data with different band combinations. The sensitivity to the training sample size and the computational efficiency of the machine learning algorithms are also evaluated. This study may provide guidance for the future work of LAI inversion.

The organization of this paper is as follows. Section 2 introduces the method to retrieve LAI from time-series MODIS reflectance data. This includes a framework for LAI inversion from time-series data using machine learning algorithms, the method to generate the training and testing samples, the machine learning algorithms used in this study and their training. The statistical parameters used to evaluate the performance of the machine learning algorithms are also briefly described in this section. Section 3 presents the performance comparison of the machine learning algorithms. The conclusions are given in Section 4.

## 2. Methodology

### 2.1. A Framework for LAI Inversion from Time-Series Data Using Machine Learning Algorithms

**Figure 1:**
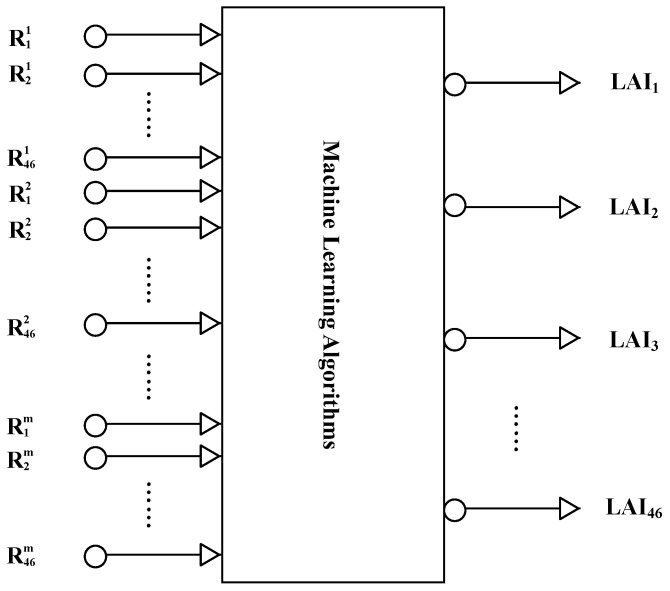
Schematic description of the method to retrieve leaf area index (LAI) values from time-series Moderate Resolution Imaging Spectroradiometer (MODIS) surface reflectance data.

In this study, machine learning algorithms are used to retrieve LAI from time-series MODIS surface reflectance data (MOD09A1). All the machine learning algorithms employ multi-input-multi-output architectures. In contrast to existing methods that only use remote sensing data acquired at a specific time to retrieve LAI, the reprocessed MODIS surface reflectance data for a one-year period were inputted into the machine learning algorithms to estimate one-year LAI profiles (Figure 1).

The MOD09A1 product has an eight-day temporal resolution. To retrieve LAI profiles from time-series MODIS surface reflectance data using the machine learning algorithms, the input vector X of the machine learning algorithms comprised the reprocessed MODIS time-series reflectance values (for a one-year period); that is, $X={\left(R_{1}^{1},R_{2}^{1},......,R_{46}^{1},R_{1}^{2},R_{2}^{2},......,R_{46}^{2},......,R_{1}^{m},R_{2}^{m},......,R_{46}^{m}\right)}^{T}$ contains 46×m components, where m is the number of bands. The output vector $Y^{\prime }={\left(LAI_{1},LAI_{2},......,LAI_{46}\right)}^{T}$ is the corresponding LAI time series for the year, which contains 46 components.

### 2.2. Generating the Training and Testing Samples

To produce GLASS LAI product from time-series MODIS/AVHRR reflectance data using GRNNs, Xiao et al. [12,24] generated a training dataset from MODIS and CYCLOPES LAI products and MODIS surface reflectance product of the BELMANIP sites during the period from 2001–2003. The effective CYCLOPES LAI was first converted to the true LAI, which was then combined with the MODIS LAI. The MODIS surface reflectance was reprocessed to remove remaining effects of cloud contamination and other factors. GRNNs were then trained using the fused time-series LAI values and reprocessed MODIS surface reflectance data over the BELMANIP sites.

The training dataset is globally representative of surface types and conditions. In this study, the dataset was used to training and testing the machine learning algorithms and evaluate their performances. Twenty groups of training samples with different sample sizes and the corresponding testing samples were randomly generated from the dataset to evaluate the sensitivity of the machine learning algorithms to the size of the training samples. Table 1 shows the numbers of training and testing samples for each group (denoted as G1 to G20). To avoid attributes in greater numeric ranges dominating those in smaller numeric ranges and numerical difficulties during the calculation, the training and testing samples were scaled before applying machine learning algorithms [25,26]. In this study, the following formula was used to normalize the values of the training and testing samples [12]:

(1) $$X_{norm}=2.0\times (X-X_{\min})/(X_{\max}-X_{\min})-1,$$

where $X_{\max}$ and $X_{\min}$ denote the maximum and minimum values for variable X, respectively, and $X_{norm}$ is the normalized value corresponding to the variable X.

### 2.3. Machine Learning Algorithms and Their Training

Four machine learning algorithms, including BPNN, radial basis function network (RBFNs), GRNNs, and MSVR, were used to retrieve LAI from time-series MODIS surface reflectance data and their performance was evaluated. The architecture of these machine learning algorithms and their training processes are as follows.

#### 2.3.1. BPNN

BPNN is named for the use of error back-propagation algorithm [27]. It is a multi-layer feedforward neutral network that is currently one of the most widely used neural network algorithms. BPNN has a simple structure and it is used mainly for pattern recognition and classification, function approximation, data compression and prediction, and other applications. This method overcomes the limitations of the perceptron and linear neural network, so it can achieve any linear or nonlinear function mapping. But in the actual operation process, it often requires repeated trials to determine the training parameters and continued training to obtain satisfactory results [27].

**Figure 2:**
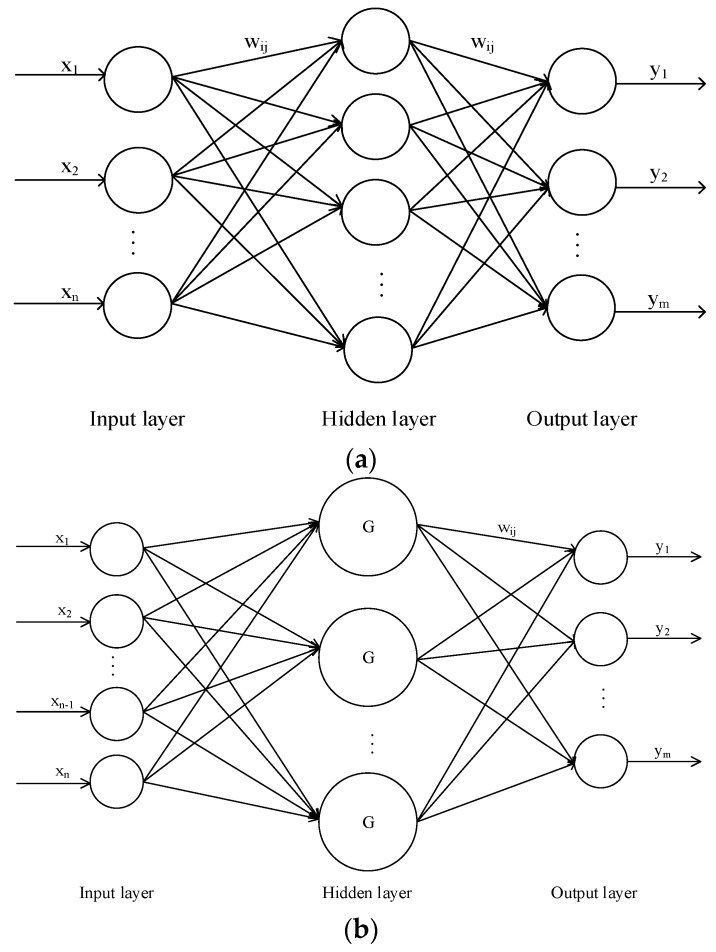

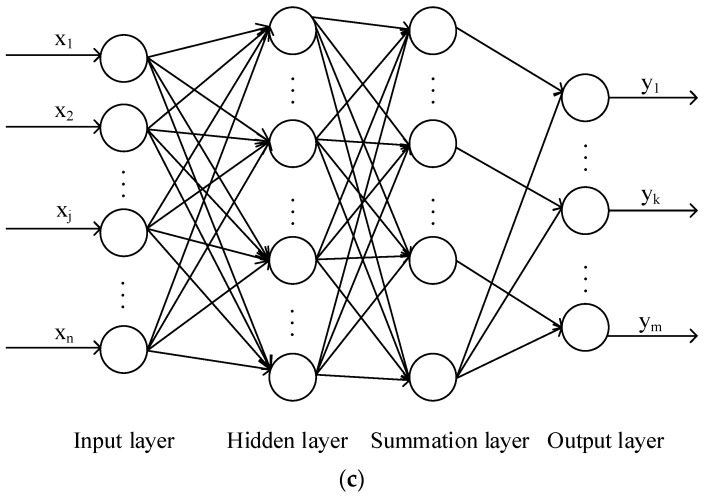
Multi-input-multi-output architectures of: (**a**) back-propagation neural networks (BPNN); (**b**) radial basis function networks (RBFNs); and (**c**) general regression neutral networks (GRNNs).

Some studies have shown that one-hidden-layer network is sufficient to model any complex system with any desired accuracy [28,29,30,31,32], so the BPNN used in this study had three layers (Figure 2a): input layer, one hidden layer, and output layer. Many investigations have analyzed the determination of the number of hidden nodes and several empirical equations have been proposed [33,34,35]. In this study, the number of neurons in the hidden layer was determined by the following equation [33]:

(2) $$n_{h}=\sqrt{n_{i}+n_{o}}+a,$$

where $n_{h}$ is the number of hidden layer nodes, $n_{i}$ and $n_{o}$ are the numbers of input and output samples, respectively, and a is a constant between 1 and 10. The input layer contained 46×m nodes and the output layer had 46 nodes, which corresponded to the one-year LAI values. Changes of learning parameters mainly affect the speed of convergence of the learning procedures, but they do not affect the BPNN structure and its overall performance [36], so the learning rate was set to 0.01. Back-propagation has disadvantages in terms of its slow convergence speed and a high likelihood of being trapped by local minima, but some improved training algorithms are available for BPNN. The Levenberg-Marquardt algorithm [37] is used widely in these training algorithms, although it occupies a large memory when applied to big training samples. The scaled conjugate gradient algorithm [38] is a normalized conjugate gradient method with low memory requirements and it is the only method that does not need linear search. Thus, the scaled conjugate gradient algorithm was used as the training algorithm in this study. In addition, the convergence criteria used for training the BPNN model comprised a mean squared error (MSE) less than or equal to 0.001 or a maximum of 5000 iterations.

The initial weights of the BPNN were set with randomly selected values. The training samples were then passed through the network and the result was outputted. Next, the network output was compared with the desired output and the error was calculated. This process is known as feed-forward. The error was back-propagated through the network and the weights of the connections were adjusted in a back-propagation process. The process of feeding forward the training samples and back-propagating the error continued until the error was minimized in the network or it reached an acceptable magnitude [39,40].

#### 2.3.2. RBFNs

RBFNs, developed by Broomhead and Lowe [41], are also feedforward neutral networks, and they have been used widely for function approximation and pattern recognition in recent years. Figure 2b shows the architecture of the RBFN used in this study, which comprises three layers: input layer, output layer, and one hidden layer. The output of a RBFN can be described by the following equation [42]:

(3) $$Y^{\prime }=w_{0}+\sum_{i=1}^{n_{h}}w_{i}f(\left\|X-c_{i}\right\|),$$

where f(⋅) represents a radial-basis function (RBF), $w_{i}$ are the output layer weights, $w_{0}$ is an offset, X are the inputs to the network, $c_{i}$ are the centers of the basis functions, $n_{h}$ is the number of nodes in the hidden layer, and ‖⋅‖ is the Euclidean norm. RBFN has a similar architecture to the BPNN, but it only has one hidden layer. The input into RBFN is nonlinear, whereas the output is linear. The weights between the input and hidden layers are 1, and only the weights between the hidden and output layers can be tuned.

The basis function used in this study is the following Gaussian basis function:

(4) $$f(X)=\exp\frac{-{\left(X-c\right)}^{2}}{r^{2}},$$

where r is the basis function width, which is the only free parameter. More details of basis functions can be found in Haykin (1999) [43].

There are two stages during the training of RBFNs. First, the basis functions are determined by unsupervised analysis, and the weights connecting the nodes in the input and hidden layers are then defined effectively. For a given input, the Euclidean norm computes the distance from the center of its RBF. If the input vector is closer to the function center, then the RBF will yield a larger number. Second, the weights connecting the hidden and output layers are derived by a linear supervised method. For a given input vector, one or more nodes will provide an output because local data are employed for modeling the RBFNs. The outputs are then linearly combined to form the network output [40,42].

#### 2.3.3. GRNNs

GRNNs, developed by Specht [44], are a generalization of RBFNs and probabilistic neural networks. At present, GRNNs are used widely for system identification, adaptive control fields, pattern recognition, and time series prediction. GRNNs comprise four layers, including the input layer, pattern layer, summation layer, and output layer. Figure 2c shows a GRNN with a multi-input-multi-output architecture. The kernel function of the GRNN used in this study is Gaussian and its fundamental formulation is as follows:

(5) $$Y^{\prime }(X)=\frac{\sum_{i=1}^{N}Y^{i}\exp\left(-\frac{D_{i}^{2}}{2\sigma^{2}}\right)}{\sum_{i=1}^{N}\exp\left(-\frac{D_{i}^{2}}{2\sigma^{2}}\right)},$$

where $D_{i}^{2}={\left(X-X^{i}\right)}^{T}(X-X^{i})$ is the squared Euclidean distance between the input vector X and the *i*th training input vector $X^{i}$, $Y^{i}$ is the output vector corresponding to the vector $X^{i}$, $Y^{\prime }\left(X\right)$ is the estimate corresponding to the vector X, N is the number of samples, and σ is a smoothing parameter for controlling the size of the receptive region. Thus, the smoothing parameter is the only free parameter.

In this study, the shuffled complex evolution method developed at the University of Arizona (SCE-UA) was used to obtain the optimal smoothing parameter when the following cost function of the smoothing parameter was minimized [12]:

(6) $$f(\sigma )=\frac{1}{N}{\sum_{i=1}^{N}\left({\hat{Y}}_{i}(X_{i})-Y_{i}\right)}^{2},$$

where ${\hat{Y}}_{i}(X_{i})$ is the estimate corresponding to $X_{i}$ using the GRNN trained over all of the training samples except the *i*th sample.

#### 2.3.4. MSVR

The support vector machine (SVM) was proposed by Vapnik [45] and has become a very popular machine learning method. It is a learning systems based on Vapnik statistical learning theory and the structural risk minimization principle, avoiding local minima and over learning problems. Support vector regression (SVR) is the application of support vector machine in function regression area. The traditional SVR algorithm only applies to single-output systems. MSVR is a multivariate regression estimation algorithm based on the SVR [20,21,22]. It expands single-output SVR into multiple-output and uses-insensitive cost function to maintain simple and sparse solution.

In the multidimensional regression estimation problems, the expected output y is a vector with Q variables to be predicted, i.e., $y\in R^{Q}$. Thus, we have to find a regressor $w^{j}$ and $b^{j}\left(j=1,\ldots ,Q\right)$ for every output in the multidimensional regression estimation problem. We can directly generalize the 1-D SVR to solve the multidimensional case by minimizing the following formulation:

(7) $$L_{P}(W,b)=\frac{1}{2}\sum_{j=1}^{Q}{\left\|w^{j}\right\|}^{2}+C\sum_{i=1}^{l}L(u_{i}),$$

where $u_{i}=\left\|e_{i}\right\|=\sqrt{e_{i}^{T}e_{i}}$, $e_{i}^{T}=y_{i}^{T}-\phi^{T}(x_{i})W-b^{T}$, $W=[w^{1},...,w^{Q}]$, $b={\left[b^{1},...,b^{Q}\right]}^{T}$, and ϕ(⋅) is a transformation to a higher-dimensional space. L(u) is the Vapnik ε-insensitive loss-function and can be expressed as:

(8) $$L(u)=\{\begin{array}{cc} 0, & u<\varepsilon \\ u^{2}-2u\varepsilon +\varepsilon^{2}, & u\geq \varepsilon \end{array}.$$

It is usual to work with the feature space kernel (inner product of the transformed vectors $k(x_{i},x_{j})=\phi^{T}(x_{i})\phi (x_{j})$), instead of the whole nonlinear mapping. In this study, the RBF kernel is used because it can handle the case when the relationship between class labels and attributes is nonlinear with mapping nonlinearly samples into a higher dimensional space. There are three parameters for MSVR with a RBF kernel: the penalty coefficient C, loss function ε and kernel width parameter σ. The SCE-UA algorithm combined with exponential transformation was used to search the optimal values of these parameters [26].

### 2.4. Evaluation Statistics

After the machine learning algorithms were trained using the training data, the testing data were used to evaluate the performance of these algorithms. The reprocessed MODIS reflectance data for a one-year period were entered into the trained machine learning algorithms and the outputs of the trained machine learning algorithms represented the one-year LAI profile for each pixel.

The MODIS surface reflectance data provide seven bands with the corresponding center wavelengths of 648 nm, 858 nm, 470 nm, 555 nm, 1240 nm, 1640 nm and 2130 nm. In this study, the MODIS reflectance in the red band (620–670 nm), near infrared band (841–876 nm) and shortwave infrared band (1628–1652 nm) were used to evaluate the performance of the four machine learning algorithms through different band combinations. The band combinations comprised one band (only red band or only NIR band), two bands (red and NIR bands), and all three bands. Meanwhile, the performance of the four machine learning methods to retrieve LAI values from NDVI was also evaluated in this study. The LAI values derived from the MODIS surface reflectance data with different band combinations using the machine learning algorithms were evaluated with the collocated testing LAI values using the following statistical parameters: the coefficient of determination (R^2^), root mean squared error (RMSE), and mean absolute error (MAE):

(9) $$\mathrm{RMSE}=\sqrt{\frac{1}{N}\sum_{i=1}^{N}{\left(LAI_{i}^{e}-LAI_{i}^{t}\right)}^{2}},$$

(10) $$\mathrm{MAE}=\frac{1}{N}\sum_{i=1}^{N}\left|LAI_{i}^{e}-LAI_{i}^{t}\right|,$$

where $LAI_{t}^{e}$ and $LAI_{i}^{t}$ represent the estimated and testing LAI values, respectively, and N is the total number of data points. Indicators similar to these are often used in performance evaluation studies [11,12]. In addition, the computational efficiency of the four machine learning algorithms was also evaluated in this study.

## 3. Results and Discussion

### 3.1. Sensitivity of the Machine Learning Algorithms to Sample Size

**Figure 3:**
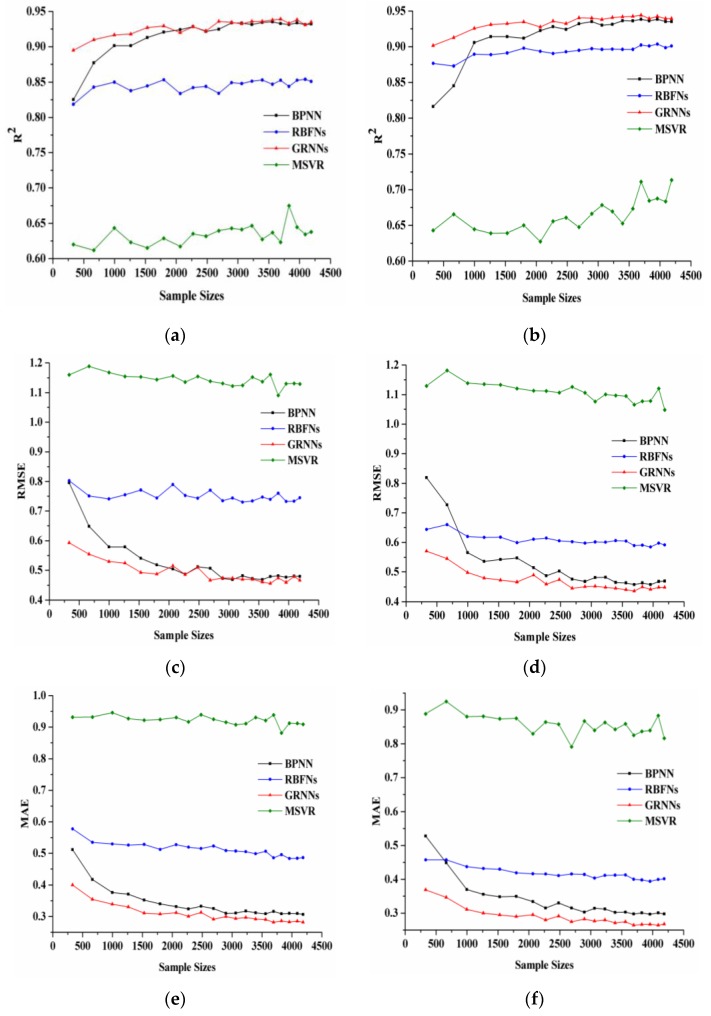
Sensitivity of the machine learning algorithms to sample size. Changes of R^2^ with training sample sizes for (**a**) the LAI values derived from reflectance data in the red and NIR bands and (**b**) the LAI values derived from reflectance data in the red, NIR, and SWIR bands; changes of RMSE with training sample sizes for (**c**) the LAI values derived from reflectance data in the red and NIR bands and (**d**) the LAI values derived from reflectance data in the red, NIR, and SWIR bands; changes of MAE with training sample sizes for (**e**) the LAI values derived from reflectance data in the red and NIR bands and (**f**) the LAI values derived from reflectance data in the red, NIR, and SWIR bands.

Figure 3 shows the changes of R^2^, RMSE, and MAE as the sample size varied. In the left panel, the LAI values were derived from the MODIS reflectance data in the red and NIR bands, whereas in the right panel, the LAI values were derived from the MODIS reflectance data in the red, NIR, and SWIR bands. With the increase of the sample size, R^2^ of the RBFNs, GRNNs and MSVR increased gradually, which indicates that the performance of the three machine learning algorithms has a slight improvement with the sample size increased. For BPNN, R^2^ increased rapidly with increasing sample size up to 2000. When the sample size was larger than 3000, R^2^ of BPNN was almost unchanged and slightly smaller than that of GRNNs. Among the four machine learning algorithms, MSVR has the smallest R^2^ for any sample size. As the sample size increased, RMSE and MAE of these four methods decreased. BPNN and GRNNs achieved significantly better performance than RBFNs and MSVR in terms of the RMSE and MAE. For BPNN, RMSE and MAE decreased rapidly with increasing sample size up to 2000. RBFNs just had smaller RMSE and MAE values than MSVR, whose RMSE and MAE values were the largest among the four machine learning algorithms.

The changes of R^2^, RMSE, and MAE for the LAI values derived from the MODIS reflectance data in the red and NIR bands were similar to those in the red, NIR, and SWIR bands. As the sample size increased, the metrics changed with slight fluctuations and become stable gradually. However, there are also some differences between the results shown in the left and right panels. First, R^2^ of all these methods using MODIS reflectance data in the red and NIR bands were smaller than those in the red, NIR, and SWIR bands, whereas RMSE and MAE of all these methods using MODIS reflectance data in the red and NIR bands were larger than those in the red, NIR, and SWIR bands. Second, BPNN and GRNNs had more similar performance in red and NIR bands than those in red, NIR, and SWIR bands.

Figure 3 shows that all of the algorithms obtained rather good performance (R^2^ > 0.61, RMSE < 1.19, and MAE < 0.95). The results show that GRNNs had the least sensitivity to the sample size and BPNN had the most sensitivity. RBFNs and MSVR were also less sensitive to the sample size, but they performed worse than the other methods.

### 3.2. Comparisons of the Retrieved LAI from MODIS Reflectance Data with Different Band Combinations

The above results showed that the retrieved LAI was more accurate when the sample size was larger. Thus, the training data with the maximum sample size was used to evaluate performance of the four machine learning methods to retrieve LAI values from MODIS reflectance data in different band combinations.

**Figure 4:**
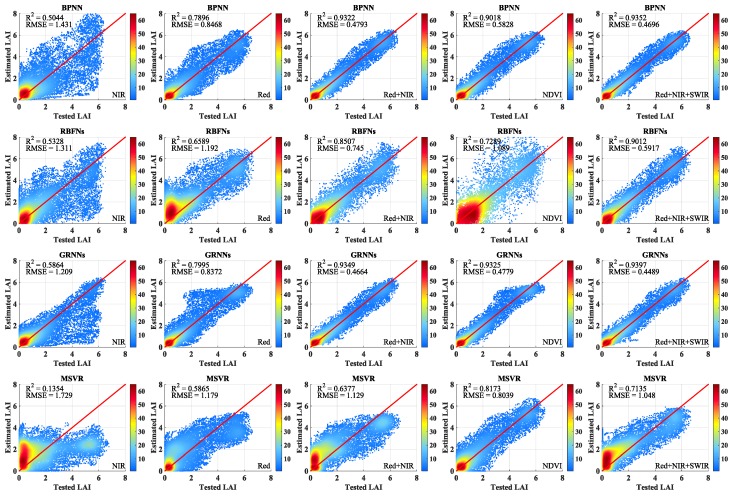
Scatter density plots of four machine learning methods between the testing LAI values and the LAI values retrieved from MODIS surface reflectance data in different band combinations and NDVI values.

Figure 4 shows the scatter density plots with different band combinations and NDVI between the testing LAI values and the retrieved LAI values using the machine learning methods. The scatter density plots between the testing LAI values and the LAI values retrieved from the MODIS reflectance data in the NIR band using the four machine learning methods are shown in the first panel in Figure 4. GRNNs performed better than the other three methods, although all four algorithms did not obtain the desired results. The second panel in Figure 4 shows the scatter density plots between the testing LAI values and the LAI values retrieved from the MODIS reflectance data in the red band. It is clear that the four machine learning methods performed better compared with the LAI values retrieved from the MODIS reflectance data in the NIR band. Among the four machine learning methods, GRNNs delivered the best performance, with the highest R^2^ and lowest RMSE values, whereas MSVR performed the worst. The third panel in Figure 4 shows the scatter density plots for the four methods between the testing LAI values and the LAI values retrieved from the MODIS reflectance data in the red and NIR bands. These results show that all of the methods performed better compared with using only one band. The fourth panel in Figure 4 shows the scatter density plots for the four methods between the testing LAI values and the LAI values retrieved from NDVI values. Performance of BPNN and GRNNs was just slightly worse than that with red and NIR bands, while the performance of RBFNs was obviously worse than that with red and NIR bands. The performance of MSVR was different with the other four methods, which performed better than that with red and NIR bands. The final panel in Figure 4 shows the scatter density plots for the four methods between the testing LAI values and the LAI values retrieved from the MODIS reflectance data in the red, NIR, and SWIR bands. The LAI values retrieved from the three bands reflectance data were slightly better compared with the LAI values retrieved from the red and NIR bands reflectance data. The LAI values retrieved from reflectance data in the red, NIR, and SWIR bands were almost the same using BPNN and GRNNs. Among the four machine learning methods, MSVR had the worst performance, with the lowest R^2^ and largest RMSE values.

Figure 4 demonstrates that all the methods performed better, with higher R^2^ and lower RMSE and MAE values, as the number of bands increased. Using only one band reflectance data to train the methods for LAI retrieval, the LAI values retrieved from surface reflectance in the red band were better than those obtained from the NIR band reflectance. All the methods performed well when MODIS surface reflectance data in the red and NIR bands were used together to train the methods. All the methods had a worse performance with the NDVI values than that with red and NIR bands, except MSVR. When the reflectance data in the red, NIR and SWIR bands were used to train the methods, the retrieved LAI values using the four machine learning methods improved further. Regardless of the band combinations employed, GRNNs outperformed the other three methods. BPNN obtained almost the same performance, whereas the performance of RBFNs and MSVR was worse than that of the other two methods.

In addition, we also note that RBFNs are affected by the problem of negative values during LAI inversion. When the testing data are at the edge of the training data range, the estimates of the RBFNs may appear the mutation. Furthermore, RBFNs based on the empirical risk minimization principle have a simple structure with only one hidden layer, which may lead to overfitting. These reasons may explain the negative values obtained by RBFNs.

### 3.3. Comparisons of Computational Efficiency

Computational efficiency is an important evaluation metric for operational algorithms to generate global LAI products. Thus, the time required by the four methods for the training and testing processes was computed to further assess the performance of these methods. The MODIS surface reflectance data in the red, NIR, and SWIR bands were used to train the machine learning methods. The computer used to execute these programs had an Intel(R) Core(TM) i7-3770K CPU @ 3.40 GHz processor and installed memory (RAM) of 8.00 GB.

**Figure 5:**
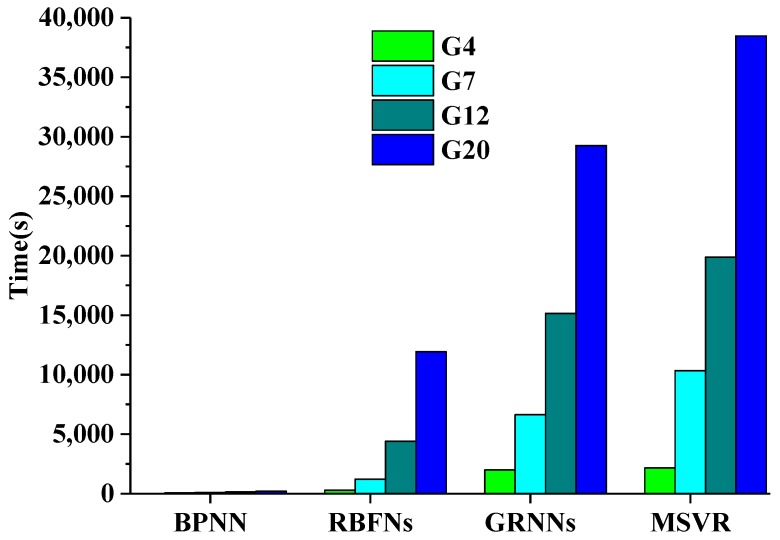
Time required for the training process by the machine learning methods with different sample sizes.

Figure 5 shows histograms of the time required under different sample sizes by these methods during the training process. BPNNs required the least time for the training process regardless of the sample size. RBFNs had a relatively fast convergence speed when the training sample size was small. RBFNs only has one hidden layer, so it needed more nodes compared with BPNN when the number of training samples was large, thereby highlighting its disadvantages in terms of efficiency. GRNNs and MSVR consumed much more time than the other two methods. MSVR consumed slightly more time than GRNNs for any sample size.

**Figure 6:**
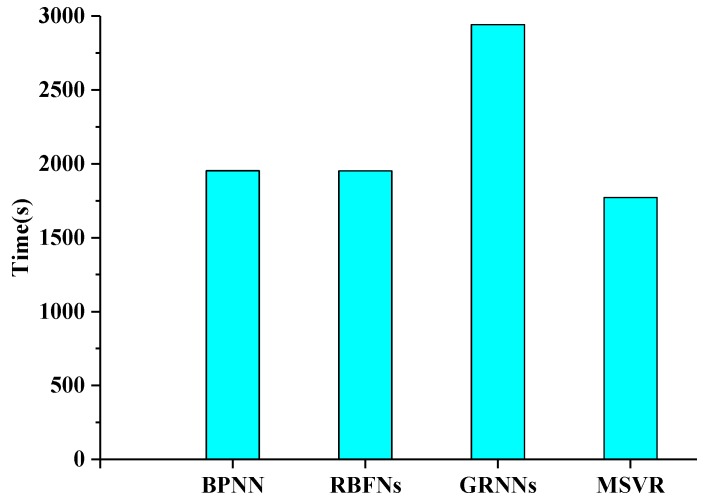
Time required using the four machine learning methods to retrieve LAI values over a MODIS tile in 1200 rows by 1200 columns.

Figure 6 shows the time required by the four machine learning methods to retrieve LAI values from MODIS surface reflectance data with red, NIR, and SWIR bands over a MODIS tile in 1200 rows by 1200 columns. These results demonstrate BPNN, RBFNs, and MSVR required nearly the same time for LAI retrieval over a MODIS tile. GRNNs required the most time compared with the other three methods.

## 4. Conclusions

The currently LAI inversion methods use remote sensing data at the specific time for LAI retrieval, which yields spatially incomplete and temporally discontinuous LAI products. In this study, four machine learning algorithms were proposed to retrieve LAI from time-series MODIS reflectance data, and performance of these machine learning algorithms was evaluated. The results showed that RBFNs, GRNNs and MSVR were less sensitive to the training sample size, whereas BPNNs exhibited high sensitivity. The BPNNs produced poor LAI estimates with small training samples. Comparison of the LAI values retrieved from MODIS surface reflectance data in different band combinations using the four machine learning methods demonstrated that all of the methods performed better as the number of bands in the samples increased. Among the four machine learning methods, GRNNs achieved the best performance irrespective of the training sample size and it also retrieved the best results regardless of the band combinations employed. When the training samples were sufficiently large, all four machine learning methods delivered improvement in LAI retrieval and BPNN performed almost the same as GRNNs. Comparison of the training time required by the four methods demonstrated that BPNN required the least time regardless of the sample size. RBFNs had a relatively fast convergence speed when the training sample size was small. GRNNs and MSVR consumed much more training time than the other two methods for any sample size. BPNN and RBFNs required nearly the same time and slightly more than MSVR for LAI retrieval over a MODIS tile. GRNNs required the most time compared with the other three methods. Considering the performance of the four machine learning methods, BPNN and GRNNs can be recommended as operational methods for LAI retrieval from time series satellite observations.

## Figures and Tables

**Table 1:** Numbers of training and testing samples in each group.

**Sample Name**	**G1**	**G2**	**G3**	**G4**	**G5**	**G6**	**G7**	**G8**	**G9**	**G10**
Training Datasets	332	664	996	1263	1530	1797	2064	2272	2481	2689
Testing Datasets	332	332	332	332	332	332	332	332	331	331
**Sample Name**	**G11**	**G12**	**G13**	**G14**	**G15**	**G16**	**G17**	**G18**	**G19**	**G20**
Training Datasets	2896	3062	3228	3394	3560	3693	3826	3959	4091	4187
Testing Datasets	332	332	332	332	332	332	332	332	332	332
